# Complex Regional Pain Syndrome in Athletes: Scoping Review

**DOI:** 10.3390/medicina57111262

**Published:** 2021-11-17

**Authors:** Antimo Moretti, Angela Palomba, Marco Paoletta, Sara Liguori, Giuseppe Toro, Giovanni Iolascon

**Affiliations:** Department of Medical and Surgical Specialties and Dentistry, University of Campania “Luigi Vanvitelli”, 80138 Naples, Italy; antimo.moretti@unicampania.it (A.M.); angela.palomba@unicampania.it (A.P.); sara.liguori@unicampania.it (S.L.); giuseppe.toro@unicampania.it (G.T.); giovanni.iolascon@unicampania.it (G.I.)

**Keywords:** complex regional pain syndromes, athlete, sport, pain

## Abstract

*Background and Objectives:* Complex regional pain syndrome (CRPS) is a chronic condition characterized by disproportionate regional pain, usually affecting distal limbs, that follows trauma or surgery. Athletes may develop CRPS because of exposure to traumatic or overuse injuries. The aim of the present study is to review the available literature about CRPS type 1 in athletes. *Materials and Methods*: We searched two online databases (PubMed and Web of Science), selecting papers aiming at investigating CRPS type 1 (algodystrophy) in athletes. The analysis of databases was made considering original articles published until 30 June 2021, written in English. *Results:* Fifteen papers (12 case reports, 3 case series) were selected for a total of 20 clinical cases (15 females, 5 males), aged between 10 and 46 years (mean age 18.4 ± 9.8 standard deviation years). Patients included practiced different types of sport (soccer, athletics, gymnastics, basketball). The most involved anatomical sites were lower limbs, and time to diagnosis ranged from 2 days to 4 years. The most used treatments were pharmacological and physical therapies, but sometimes invasive approaches, as regional nerve, or lumbar sympathetic blocks, were provided. The main assessed outcomes were return to activity and pain. *Conclusions*: Our review suggests a higher prevalence of CRPS type 1 in younger people and in lower limbs than in general population but confirms the higher prevalence in females. However, the number of studies addressing CRPS in athletes is limited, as well as the number of involved patients, considering that only few and heterogeneous case reports were published about this topic. Moreover, the high prevalence of old studies (only 5 available studies in the last 10 years) might have influenced the choice of both assessment tools and management strategies. Despite these limitations, athletes showing disproportionate pain after sport-related injury should be promptly evaluated and treated through a multidimensional approach to avoid long-term consequences of algodystrophy.

## 1. Introduction

Complex regional pain syndrome (CRPS) is a rare clinical condition, usually occurring after appendicular trauma or surgery, characterized by extremely variable signs and symptoms of the affected limb [1,2]. The main CRPS patients’ complaint is continuing pain, often burning, that is disproportionate to its underlying cause, usually accompanied with sensorimotor (muscle weakness, tremor, dystonia, hyperesthesia, and/or allodynia), vasomotor (temperature and color changes of skin), sudomotor (edema and/or sweating), and trophic changes of the affected site, and often localized at the upper or lower limb extremities [1,3]. Nowadays, different forms of CRPS, with overlapping clinical features, have been defined [4]: CRPS type I (algodystrophy), CRPS type II (causalgia), CRPS not otherwise specified (NOS), and CRPS with remission of some features (CRSF). In algodystrophy, clinical findings have a non-dermatomal pattern (regional) in the distal region of the affected limb, while causalgia can develop after a clearly detectable nerve injury. CRPS-NOS partially reproduces the clinical scenario of other forms, and it is not better explained by any other condition. CRSF is a new type of CRPS, with partial remission, whose characteristics are still not well defined [4].

CRPS is considered among the most painful diseases even though the causes and pathogenic mechanisms of pain are mostly unknown [5,6]. CRPS can occur after crash injuries, fractures, or surgery, but in younger people it can follow minor accidents, as strain, sprain, or bone bruise [7].

According to presentation of all CRPSs, it has been described as having two phenotypes, inflammatory or warm and chronic or cold form. Current diagnosis of CRPS type I is based on clinical features (Budapest criteria) [8], while the role of imaging techniques is still debated [9].

Considering that the precipitating event in CRPS is often represented by an injury, such as fractures or sprains, this condition could be relevant for athletes, even if a direct relation between sport activity and CRPS risk has not been defined [7]. Indeed, in this context, sport-related injury could be a driving cause of CRPS in young people, due to trauma and/or aberrant exaggerated inflammatory processes. Athletes experiencing worsening conditions after common trauma should be assessed for excluding CRPS [10]. The intensity and frequency of sport activity may be linked to augmented risk of injury and CRPS. To the best of our knowledge, a comprehensive review on CRPS in sport practice is not available so far. The aim of the present study is to review the available literature about algodystrophy (CRPS type I) in athletes.

## 2. Materials and Methods

We searched two online databases: PubMed (PM) and Web of Science (WoS). The selection of articles was made through the following search string: (“Athlete” OR “Sport” OR “Player”) AND (“Complex Regional Pain Syndromes” [Mesh] OR “Algodystrophy”). Moreover, we checked the reference list of all the screened full-text articles.

The analysis of databases was made through the following criteria: (i) articles published from inception until 30 June 2021; (ii) original articles, excluding reviews, commentaries, posters, and proceeding papers; (iii) only full paper written in English. After applying the research process (A.P.), two authors (A.M. and A.P.) independently reviewed the titles and abstracts of available articles to check the matching with the research aim and inclusion criteria. They selected papers aiming at investigating CRPS type 1 in athletes and combined the articles obtained from the two databases, excluding duplicates. Single-case studies, case series, and cohort studies were selected. After full text reading, they excluded (i) articles dealing with CRPS type 2, NOS or CRSF; (ii) review articles; (iii) articles dealing with patients not practicing any sport at any level. Moreover, additional papers matching the inclusion and exclusion criteria were found by screening the reference list of the articles found through the research process.

From the selected papers, the following data were extracted: (i) author(s) and year of publication; (ii) participant characteristics (number, age, sex); (iii) sport practiced; (iv) time to diagnosis; (v) affected site; (vi) comorbidity; (vii) treatment; (viii) outcome(s).

## 3. Results

**Figure 1:**
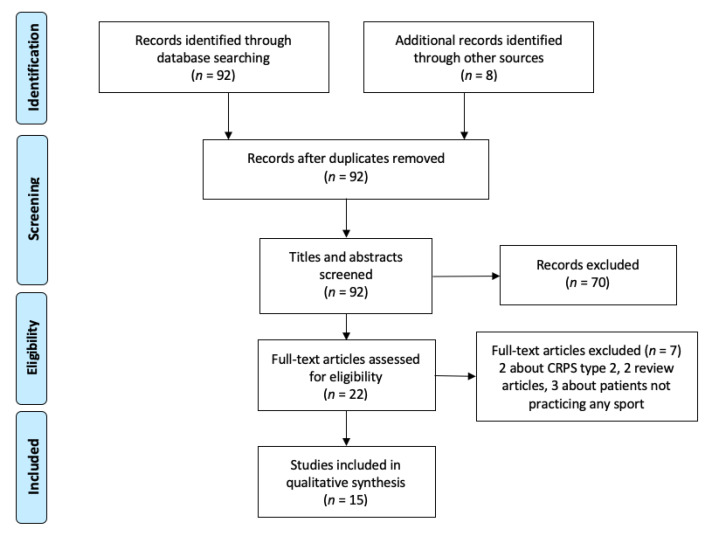
Flow diagram of the literature review process.

The review process results are shown in Figure 1, according to the PRISMA guidelines for scoping reviews (PRISMA-ScR) [11].

After applying the paper selection criteria, we checked 21 full texts and excluded 2 articles dealing with CRPS type 2, 2 review articles, and 3 articles dealing with patients not practicing any sport. Finally, the selected articles were 15. Table 1 shows main characteristics of each study.

Most of the available studies are case reports. Indeed, only three studies involve, respectively, 2 and 3 patients, for a total number of 20 patients. The 3 patients from Ladd et al. [17] were extracted from a cohort of 11 patients, 4 of whom were not practicing any sport and the others were excluded for data unavailability. The selected studies were published from 1989 to 2021. Sex prevalence is in favor of females (15 F: 5 M). The participants’ age ranged from 10 to 46 years (mean age 18.4 ± 9.8 standard deviation years). The patients practiced different types of sport: soccer (5 studies), athletics or running (5 studies), hockey (3 studies), gymnastics (2 studies), basketball (2 studies), volleyball (2 studies), swimming, triathlon, baseball, handball, powerlifting, and wrestling. Table 2 provides detailed information about the selected studies. Only two studies [14,15] followed Budapest diagnostic criteria. Imaging, including X-ray, ultrasound, magnetic resonance, computer tomography, and bone scans, was often used for differential diagnosis. Only four studies reported details about warm- [20,21,26] or cold-type [16] CRPS in the considered clinical case.

Time to diagnosis varies from a few days to several months, reaching up to 4 years in Hind et al. [15]. The most frequent triggering events were sprains, while CRPS presentation timing from trauma even varied from days to months. The most involved site was the lower limb, including calf, knee, ankle, and foot. Only one study [23] reported a wrist involvement. Athletes often presented previous traumatic or overuse injuries, clinical conditions (such as osteopenia or amenorrhea, or depression) that could be directly or indirectly (e.g., predisposing to stress fractures [14]) trigger CRPS, or other comorbidities (such as type 1 von Willebrand disease, or migraine). It is noteworthy that a single study reported Calve–Perthes disease as a comorbidity [15], which likely contributed to poor bone strength and lean mass. Treatments included drugs (gabapentin, pregabalin, tricyclic antidepressants, selective serotonin reuptake inhibitors, steroids, opioids, or local medication with lidocaine), physical therapy (including desensitization techniques and transcutaneous electrical nerve stimulation (TENS)), occupational therapy (OT), psychological counseling, regional nerve blockade (RNB) using ketorolac and lidocaine or ropivacaine and clonidine, and lumbar sympathetic block (LSB) using bupivacaine or guanethidine. Invasive approaches, such as RNB and LSB, were used to treat patients unresponsive to non-invasive therapies and to facilitate the execution of physical therapy when it was limited by pain. The main outcomes used to evaluate treatment response were joint range of motion (RoM) of the affected joints, gait parameters (pattern, distance, and assistive device needs), weight-bearing tolerance, symptoms (most of all pain), and return to activity. The investigated treatments showed positive effects on the reported outcomes, even if with different timing. Moreover, Hind et al. [15] revealed a possible contribution in CRPS diagnostic investigation by dual-energy X-ray absorptiometry to highlight regional body composition differences. The authors found reduced bone strength and lean mass in the affected region compared to the unaffected limb and with age-matched pairs, showing lower Z-scores. This may be also due to a long-lasting CRPS with non-use of the affected region.

## 4. Discussion

Even if large epidemiological studies of CRPS type 1 in athletes are not available, it seems that this condition has different characteristics from those of the general population. CRPS has a higher incidence between 60 and 70 years [27], particularly affecting older people after surgery, fractures, or other traumatic injuries, while it seems to mostly affect young people in reported cases, probably due to the higher incidence of sport-related injury in this population [7,28]. Even if previous studies showed that in general population CRPS incidence in young people is lower than that of adults (1.16/100,000 vs. 26.2/100,000) [27,28], in the present study, apart from four studies [14,15,17,21], most of the participants ranged from 10 to 18 years. As for sex distribution, the higher prevalence in females confirms previous findings about the epidemiology of CRPS in both adults and children [9,27,28]. As regards the involved site, the upper extremity is more frequently affected than lower one [27] in the general population, while almost all involved regions are in the lower limb in athletes. This may be explained by the higher prevalence of lower limb injuries (as sprains, fractures, bruise) in sport practice [29,30]. Different comorbidities were found in the described clinical cases. Some of them, as menstrual alterations, or migraine, have already been found as predisposing factors for CRPS [14,31]. Other conditions, such as psychiatric comorbidities, are often found in CRPS patients, but their relationship has not been clarified yet [32]. Moreover, pathogenic hypothesis has been done to link other comorbidities to the CRPS occurrence, such as microvascular damage in von Willebrand disease [16].

Even if sport-related injuries are suggested as inciting event in patients with CRPS, we cannot define more hazardous ones for developing CRPS, because of the scarcity of literature to corroborate their role as risk factor.

It is worth noting that only two studies followed Budapest criteria for CRPS diagnosis and that imaging was often used for confirming diagnosis or excluding other pathologic conditions. Moreover, a clear definition of the cold or warm subtypes of CRPA cannot be found in most of the included papers.

As for the general population, the delayed diagnosis of CRPS might be a crucial issue for athletes, too. A person can procrastinate even for years before achieving a correct CRPS diagnosis, as reported also in our review. This is important, particularly in athletes, because a delayed diagnosis can lead to a worse therapeutic response and prognosis [33], thus implying a late or incomplete return to sport activity.

In the studies included in our review, pain and physical function were mostly assessed, while emotional well-being, the participants’ ratings of global improvement and satisfaction, and adverse events were not investigated [34]. Positive effects on the reported outcomes were obtained by treatment administration with different timings. Considering the huge variability in clinical scenarios and treatment response of patients with CRPS, the management of this condition should be based on a bio-psycho-social model [35] through a comprehensive assessment of impairments and activity limitations to guide multimodal interventions, including pharmacological and non-pharmacological treatments.

Concerning the treatment options for athletes developing CRPS, pharmacological therapy was often combined with physical therapy. It should be underlined that no study reported bisphosphonate use in this population (e.g., neridronate), considering that this drug class seems effective in the management of algodystrophy and is supported by moderate quality of evidence [36,37]. As for physical therapy, athletes with CRPS were treated with desensitizing techniques, early mobilization, and TENS. Early and progressive mechanical loading by avoiding muscle wasting and bone loss due to non-use could represent a key point in CRPS rehabilitation, especially in athletes [15].

The main limitations to provide reliable conclusion are the limited number of studies addressing CRPS in athletes as well as the limited number of involved patients. Moreover, the high prevalence of old studies (only 5 available studies in the last 10 years) might have influenced the choice of both assessment tools and management strategies.

## 5. Conclusions

The available findings show that CRPS can be found in young athletes. Physicians should investigate clinical findings characterizing this condition in the context of a sport-related injury with pain disproportionate to its cause, even in young people, as an early diagnosis influences the effectiveness of interventions and the prognosis. However, the best treatment to minimize symptoms and to allow a fast but safe return to activity in athletes with CRPS is not well established. Indeed, return to activity in athletes should be careful, so that tissue recovery is reached before starting activity, to avoid relapses, but it should be started as soon as possible, as the progressive stimuli and weight bearing could represent therapeutic strategies for CRPS. Future studies may focus on the comparison or combination of different types of treatments to assess which one could maximize benefits. In our opinion, an interdisciplinary and multidimensional management should be proposed to athletes with CRPS to allow adequate pain relief and promote early and safe return to play.

## Figures and Tables

**Table 1:** Main characteristics of selected articles.

Author and Year	Number of Patients	Gender	Age	Sport Practiced	BC	Imaging	Cold/Warm Type	Site
Carayannopoulos et al., 2009 [12]	1	F	12	Soccer, basketball, field hockey	No	US	n.a.	Ankle
Collins, 2007 [13]	1	M	13	Baseball, soccer, handball, basketball	No	XR, MRI, BS	n.a.	Ankle
Feldman et al., 2009 [14]	1	F	37	Triathlon	Yes	MRI	n.a.	Lower extremity
Hind et al., 2014 [15]	1	M	29	Powerlifting	Yes	DXA	n.a.	Leg
Khadavi et al., 2014 [16]	1	F	17	Athletics	No	MRI	Cold	Calf
Ladd et al., 1989 [17]	3	1 M, 2 F	18, 20, 31	Athletics, swimming, hockey	No	No	n.a.	Ankle and knee
Martìnez-Silvestrini et al., 2006 [18]	3	F	11, 13, 14	Athletics, Volleyball	No	XR	n.a.	Foot, ankle, knee
McAlear et al., 2021 [19]	1	F	18	Soccer	No	No	n.a.	Foot
Middlemas, 2007 [20]	1	F	10	Soccer	No	XR, US	Warm	Foot
Myers, 2013 [21]	1	F	46	Running	No	XR	Warm	Knee
Rand, 2009 [22]	1	F	10	Gymnastics	No	MRI	n.a.	Knee
Suresh et al., 2002 [23]	2	F	11–15	Gymnastics, volleyball	No	No	n.a.	Foot and wrist
Takahashi et al., 2018 [24]	1	M	12	Soccer	No	XR, CT	n.a.	Ankle
Walia et al., 2004 [25]	1	M	13	Wrestling	No	XR, MRI, BS	n.a.	Ankle
Weber et al., 2002 [26]	1	F	18	Field hockey	No	XR, BS	Warm	Ankle

Abbreviations. BC: Budapest criteria; BS: bone scan; CT: computer tomography; MRI: magnetic resonance imaging; n.a.: not available; US: ultrasound; XR: X-ray imaging.

**Table 2:** Detailed information of the selected studies, including time to diagnosis, site, comorbidity, treatment, and outcomes.

Authors and Year	Time from Inciting Event	Time to Diagnosis	Comorbidity	Treatment	Main Findings
Carayannopoulos et al., 2009 [12]	Unknown time after ankle sprains	2 years	Not reported	P, PT, OT, CBT, RNB	Pain relief, increased ankle RoM and functional independence
Collins, 2007 [13]	15 months from ankle sprain	2 months	Not reported	P, PT	Pain relief, improvement of gait cadence and pattern, endurance, weight bearing tolerance, ankle RoM and strength (+)
Feldman et al., 2009 [14]	6 weeks after femoral fracture	6 weeks	Osteopenia, amenorrhea, depression	P, PT, LSPB	Reduced discomfort, normalization of local color and temperature
Hind et al., 2014 [15]	Years after orthopedic surgery	4 years	Calve–Perthes disease	LSPB, P, SCS	Not reported
Khadavi et al., 2014 [16]	Months after gastrocnemius strain	6 months	Type 1 von Willebrand disease	P, PT	Improvement of passive RoM (knee extension and ankle dorsiflexion) and gait distance, reduced device usage and increased weight-bearing tolerance
Ladd et al., 1989 [17]	3 months after ACL reconstruction; weeks after overuse; 10 days after ankle sprain	10 days–3 months	Sprain and osteoarthritis	LSPB, P, PT	Return to activity (3–27 months)
Martìnez-Silvestrini et al., 2006 [18]	1 day after ankle sprain; 3 days after overuse; 2.5 months after ankle sprain	2 days–2.5 months	Depression	P, PT	Reduced edema and pain, improvement of RoM
McAlear et al., 2021 [19]	2 weeks after tarsal tunnel release surgery	2 weeks	Depression	LSPB	Return to activity
Middlemas, 2007 [20]	No leading cause	2–3 weeks	Not available	P, PT	Improvement of weight bearing tolerance and independence in ADL, return to activity
Myers, 2013 [21]	No leading cause	10 days	Not available	P, PT	Pain relief and increased RoM
Rand, 2009 [22]	7 weeks after knee injury	6 weeks	Migraine	PT, LSPB, P, CBT	Return to activity (8 weeks)
Suresh et al., 2002 [23]	1 year after metatarsal avulsion; 2 months after wrist injury	2 months–1 year	Not available	P, PT, RNB	Return to activity (3 months), pain relief
Takahashi et al., 2018 [24]	5 days after ankle sprain	10 days	Not available	P, PT	Return to activity (35 days), pain relief
Walia et al., 2004 [25]	Unknown time after ankle sprain	Not known	Not available	P, PT, LSPB	Pain relief and gait improvement
Weber et al., 2002 [26]	16 days after ankle sprain	1 month	Not available	PT, LSPB	Improvement of symptoms, return to activity (2 months)

Abbreviations. ADL: activities of daily living; CBT: cognitive behavioral therapy; LSPB: lumbar sympathetic plexus blocks; OT: occupational therapy; P: pharmacological treatment; PT: physical therapy; RNB: regional nerve blockade; RoM: Range of Motion; SCS: Spinal Cord Stimulation.

## Data Availability

Not applicable.
